# Experimental Challenges for Reduced Genomes: The Cell Model *Escherichia coli*

**DOI:** 10.3390/microorganisms8010003

**Published:** 2019-12-18

**Authors:** Masaomi Kurokawa, Bei-Wen Ying

**Affiliations:** Graduate School of Life and Environmental Sciences, University of Tsukuba, 305-8572 Ibaraki, Japan; kurokaw8@gmail.com

**Keywords:** genome reduction, growth rate, experimental evolution, minimal genome, *Escherichia coli*

## Abstract

Genome reduction, as a top-down approach to obtain the minimal genetic information essential for a living organism, has been conducted with bacterial cells for decades. The most popular and well-studied cell models for genome reduction are *Escherichia coli* strains. As the previous literature intensively introduced the genetic construction and application of the genome-reduced *Escherichia coli* strains, the present review focuses the design principles and compares the reduced genome collections from the specific viewpoint of growth, which represents a fundamental property of living cells and is an important feature for their biotechnological application. For the extended simplification of the genomic sequences, the approach of experimental evolution and concern for medium optimization are newly proposed. The combination of the current techniques of genomic construction and the newly proposed methodologies could allow us to acquire growing *Escherichia coli* cells carrying the extensively reduced genome and to address the question of what the minimal genome essential for life is.

## 1. Introduction

Genomic engineering is making remarkable progress. Genome is not only freely edited [1,2], but is also becoming freely synthesizable upon the synthetic design [3]. The bottleneck of genomic engineering is no longer due to technical difficulties but the understanding of the genome itself. The first genomic sequence of *Escherichia coli* (*E. coli*), a representative model organism in microbiology, was determined more than 20 years ago [4]. However, the molecular and physiological functions of the whole genomic sequence remained unclear. Although the prediction of the cellular activity and the population dynamics (growth, metabolism, *etc.*) has been achieved according to the genetic information [5], the simulated consequences were different from the real cell. As the simple *E. coli* genome is still too complex to be fully explained, efforts have been made to reduce the complexity of *E. coli* genomes, i.e., removing the redundant genomic regions.

In this review, we comprehensively introduced the genome-reduced *E. coli* strains constructed so far. The experimental methods used for the genetic construction of the genome-reduced strains have been previously reviewed in detail [6]. Here, we made an effort to review the relationship between the genome reduction and the growth rate of *E. coli*, because the growth rate was one of the most important global parameters, which quantitatively represents the activity of living cells. Our previous study showed that the accumulation of the genome reduction caused a correlated decrease in the growth rates of *E. coli* [7]. This finding indicated that the growth rate was a key factor not only in determining the importance of genome size but also in understanding the size–survival relationship. Although a decrease in growth rates of genome-reduced strains has been commonly observed [8], neither the quantitative growth assay nor the specific discussion on the growth of reduced genomes has been conducted sufficiently. A thorough overview of the reduced genomes, associated with the growth properties, could provide us with valuable hints of how to construct the fast growing *E. coli* carrying the reduced genome. Here, we mainly summarized the reduced genomes in the point views of the design principles, growth, and other phenotypic features, if applicable. In addition, we proposed experimental approaches of how to achieve genome reduction without a growth decrease.

## 2. Genome Reduction in Synthetic Microbiology

### 2.1. E. coli as a Representative Cell Model

*E. coli* has been used as an important and common cell model for genetic and genomic engineering, which has benefited from the organisms’ fast growth and ease of genetic manipulation. *E. coli* has a few nutritional requirements [9] and divide at a very fast rate, exhibiting a 20-min doubling time in nutrient-rich conditions [10]. Since the discovery of restriction enzymes [11], genotypes have become highly operable. *E. coli* has a relatively high efficiency of transformation due to its ease of plasmid integration [12,13] and phage infectivity [14]. Following the successful transformation of a plasmid vector into *E. coli* in 1973 [15], intensive studies have been carried out to incorporate foreign genes into *E. coli* for high levels of gene expression [16,17,18]. Recently, innovative technologies, such as CRISPER-Cas9, have been put into practical use for genome engineering of *E. coli* [19], as well as systematic strategies were developed for protein production [20]. Taken together, *E. coli* is not only a representative cell model but also a powerful cell factory.

As a model organism, *E. coli* has been studied intensively. The first fully sequenced genome was the wild type *E. coli* strain K-12 MG1655 [4] in 1997. Although 20 years have passed, both the essential or substantial genetic requirements for *E. coli* are still unclear. Followed by the genome sequencing of another wild type *E. coli* strain W3110 [21], the molecular functions of approximately 54.1% of the total genes have been experimentally confirmed [22]. So far, the molecular functions of approximately 63.7% of the total genes in *E. coli* have been experimentally confirmed, nevertheless, approximately 28.1% still remain unpredicted [23]. Even if all the gene functions were identified, it was still difficult to fully explain the dynamics (e.g., growth) of *E. coli*, because of the complexity of the genetic and metabolic networks in the cell [24,25]. Therefore, in order to understand the whole cell as a living system, not only the construction of genetic motifs but also the simplification of genetic composition are required [26,27].

### 2.2. Genome Reduction Provides Ideal Cellular Conditions for Synthetic Design

Remarkable progresses have been made in both the technology and knowledge of synthetic microbiology for industrial applications [28]. Microbiologists have faced the problem that artificial gene circuits and metabolic pathways did not work properly as designed in vitro when they were implemented in vivo [29]. These problems might be caused by the complex genomic background, for example, the heterologous genes in the designed circuit must have been affected by the intrinsic genomic sequence. To solve the problem, a simpler cellular environment of less genetic information was required for predictable control [30]. Reducing the unessential genes and/or metabolic pathways was expected to improve the controllability of the genetic circuits [31]. Although the genome reduction studies were conducted using varied bacterial model organisms (e.g., *E. coli* [6], *Bacillus subtilis* (*B. subtilis*) [32], *Corynebacterium glutamicum* [33], *Pseudomonas putida* [34]), *B. subtilis* and *E. coli* were most studied [35], owing to the easy manipulation for genetic engineering and the fruitful databases of genomic information [23,36]. Apart from genome reduction, chemical synthesis of genomes, a so called bottom-up approach, was also being used as an approach to build a controllable cell. The most influential bottom-up achievement would be the construction of JCVI-syn1.0, *Mycoplasma capricolum* living on the synthesized *Mycoplasma mycoides* genome [37]. The bottom-up approach for bacteria was firstly used for *Mycoplasma*, which has the smallest genome among cultivatable bacteria. The success in synthetic construction of the reduced genome of *E. coli* strain MDS42 has recently been reported [38]. The approach of genome reduction is a well-established methodology and has been widely performed with *E. coli*. To date, several genome-reduced *E. coli* strains have been constructed to acquire controllable cells suitable for bioengineering [39]. The genomic regions that have played a role in adaptation to external disturbance, such as stress response genes [40,41], insertion sequence (IS) elements [42] and transposons [43], seem to be unnecessary for cells cultured in a stable environment in the laboratory. In particular, the ISs and transposons might cause unexpected mutagenesis and be troublesome in genetic engineering by unfavorable genomic mutation [44,45]. Additionally, the retention of these unnecessary genes was thought to increase the cost of DNA replication [46,47], as well as the expression of unnecessary genes can be a burden on microbial growth [48]. Removing these redundant genomic regions might result in cells holding a clean genetic background and simple metabolic pathways. It was supposed to be advantageous for the proper output in vivo that corresponds to the synthetic design in vitro.

## 3. Collections of Reduced Genomes

**Figure 1:**
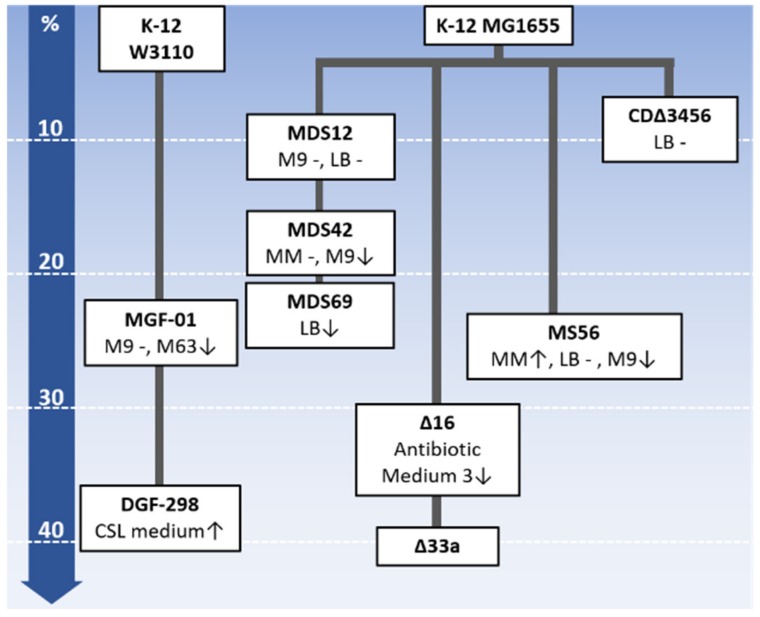
An overview of the genome-reduced *E. coli* strains. The strains are arranged from top to bottom according to decreasing genome size. The deletions responsible for the percentages in genome size reduction are indicated. The strain name, growth property and medium used are summarized within the boxes. The historical relations among the reduced genomes are connected by bold solid lines. The changes in growth rate are denoted with upward arrows, hyphens and downward arrows, which indicate increased, unchanged and decreased growth rates, respectively. The media used for the growth assay are indicated. The media (LB, MM, M9, M63, CSL medium and Antibiotic Medium 3) used for the growth assay are indicated. The compositions of the media are summarized in Table S2.

Genome-reduced *E. coli* strains have been constructed upon various concepts. Since the details of the genetic construction have been reviewed previously [6], we mainly reviewed the design principles and the growth properties (Figure 1, Table S1).

### 3.1. Minimum Genome Factory (MGF)

#### 3.1.1. Design Principles

MGF was defined as the strain that has only a set of genes necessary for fermentative production and has higher fermentative production capacity than the wild type. MGF-01, which lacked 1.03 Mb (22%) of genomic regions, was constructed based on W3110 [49]. The target regions for deletion were determined as the genomic regions in which 10 or more dispensable genes were continuously located. The dispensable genes were determined by the comparative genomic analysis between *E. coli* and *Buchnera sp*., an insect symbiotic bacterium that is divergent from the common ancestor of *E. coli* [50]. The genes that were only coded in the genome of *E. coli* were thought to be dispensable. Comparing the two genomes, a total of 95 candidate genomic regions were identified, which led to the draft design of a deduced genome with a size of 2.6 Mb. According to the draft design, 53 deletions were combined in one strain via 28 cycles of deletion transfer. MGF-01 series were constructed in consequence. To achieve a reduced genome of higher growth fitness, DGF-298, a 2.98 Mb genome, was subsequently constructed from MGF-01 [51]. The candidates for reduced genomic regions were the functionally unknown genes, harmful genes (for example ISs, prophages, and toxin-antitoxin systems), and the genomic region unique to W3110 revealed by comparative genomic analysis with pathogenic *E. coli*.

#### 3.1.2. Growth

**Figure 2:**
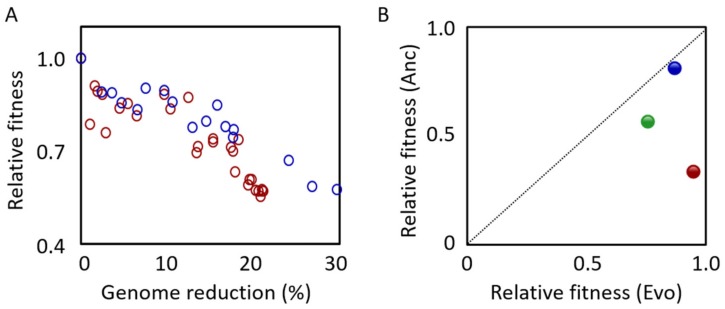
Growth decline and growth recovery of reduced genomes. (**A**). Correlation between the growth rate and genome reduction. The ratio (%) of the reduced fragment relative to the wild-type genome is indicated. Relative fitness indicates the ratio of the growth rate of the cells with reduced genomes relative to that of the parent strain cells (wild-type genome). The red and blue circles represent the reduced genomes of the MGF-01 and Δ16 derivatives, respectively. The plot was made using the data sets of the previous reports [7,8]. (**B**). Increased growth fitness attributed to experimental evolution. The relative fitness before (Anc) and after (Evo) experimental evolution are shown. Blue, green and red stand for the genome-reduced strains of MDS42, MGF-01 and MS56, respectively. The growth data was cited from previous studies [55,56,57].

The growth of MGF-01 was similar to that of W3110 in the minimum medium M9 [52,53]. In addition, MGF-01 continued to grow even after W3110 reached the stationary phase, and the saturated cell density was approximately 1.5-fold higher than that of W3110 [53]. However, our previous study showed that the exponential growth rate and the saturated cell density of MGF-01 were lower than that of W3110 in the minimum medium M63 [54], which was evaluated using 96-well microplates [7]. Intriguingly, the decrease in the growth rate was correlated with the reduced length of the genome (Figure 2A, red), and the correlation was more significant under poor nutritional conditions [7]. When MGF-01 was further reduced to 2.98 Mb (DGF-298), both the exponential growth rate and the saturated cell density remained regular [51]. It was assumed that the downregulation of the genes encoding chaperones and proteases might contribute to the growth increase [51]. Additionally, MGF-01 showed 2.4-fold higher L-threonine accumulation than W3110 [53], which was consistent with the initial design hypothesis, that is, the simpler genome resulted in higher productivity.

### 3.2. Multiple-Deletion Series (MDS)

#### 3.2.1. Design Principles

The purpose of constructing MDS was to create a “tabula rasa” strain practical for introducing foreign genes; the MDS collection was designed to have genetic stability and good metabolic efficiency by removing the ISs from the genome. The first reported strain of the MDS collection was MDS12 [58], which lacked all 12 K-islands, the specific genome region in the K-12 strain, which is equivalent to 8.1% of the original genome. The additional deletions of IS elements were performed toward MDS12, resulting in the smaller genomes of MDS41. The additional deletions of IS elements were performed toward MDS12, resulting in the smaller genome of MDS41. An endonuclease gene of *endA* was deleted from MDS41, resulting in the strain of MDS42, and the additional deletion of 45 kb from MDS42 resulted in MDS43. The genome sizes of MDS41, MDS42 and MDS43 were 14.28%, 14.30% and 15.27% smaller than the wild type genome, respectively [59]. The target regions for deletion were genomic regions found only in the K-12 strains and not in the strains CFT073, EDL933, RS218, DH10B, O157:H7, and *Shigella flexneri* 2457T [59]. By genome sequence comparison, ~ 100 genomic regions, corresponding to 20% of the entire genome and ~ 900 genes were proposed to be deleted. Furthermore, 26 additional deletions were performed for MDS43, resulting in the smallest genome MDS69 [60], in which a total length of 939.5 kb (20.3%) of the MG1655 genomic sequence was absent. The candidate regions for deletion were decided by the comparative genomic analysis between MG1655 and its close relatives (*E. coli* CFT073, EDL933, RS218, DH10B, and *Shigella flexneri* 2457T). In addition, the genes of unknown functions or coding the surface structures and mobile genetic element were targeted.

#### 3.2.2. Growth

No significant differentiation in the growth rates of MDS41 and MG1655 was detected in the minimal medium [59]. In addition, the growth rate of MDS42 was equivalent to that of MG1655 in both the minimum medium MOPS [61] and the rich medium LB [59]. However, another study reported that the growth rate of MDS42 was 20% lower than that of MG1655 in M9, which was thought to be caused by an increasing proportion of nongrowing cells in the MDS42 population [57]. However, the engineered MDS42 (MDS-205) produced 83% more L-threonine than the engineered MG1655 [62], which was consistent with the practical metabolic application as in MGF-01 [53]. A comprehensive analysis of a total of 69 MDS strains showed that the genome reduction had no advantage for growth fitness, because the growth rate of MDS69 was 17% lower than that of MG1655 [60]. The reason why the growth rate lowered was unclear. It was probably attributed to the large fluctuation in transcriptome caused by genome reduction [63,64].

### 3.3. Δ16

#### 3.3.1. Design Principles

Δ16 was constructed for the purpose of searching for a minimum set of genes sufficient to maintain the cellular functions. It was constructed based on MG1655 and lacked 1.38 Mb (29.7%) of the parent genomic sequence [8]. The genes were first classified into “essential”, “non-essential” and “unknown”, and 163 candidate regions for deletion were determined in accordance [8]. A total of 75 single-deletion mutants and 16 large deletion mutants were constructed. Δ16 was constructed by integrating the 16 target regions into one strain.

#### 3.3.2. Growth

In comparison to MG1655, Δ16 grew relatively slowly in Antibiotic Medium 3 [8]. The doubling time of MG1655 was 26.2 min, whereas it was delayed to 45.4 min for Δ16, which might be related to the changes in the cell shape, as Δ16 became thick and elongated [8]. No decline in growth was observed in the strains with a single deletion region; nevertheless, the combination of multiple deletion regions caused the growth to decrease. Intriguingly, the changes in growth seemed to be correlated with the genome reduction (Figure 2A, blue), which was similar to the result found in the MGF series. These findings indicated that the correlation between genome reduction and growth fitness was a general trend.

### 3.4. MS56

#### 3.4.1. Design Principles

MS56 was also constructed based on MG1655 and lacked 23% of the original genomic regions [65]. This strain was constructed for the purpose of acquiring a highly stable cellular host capable of producing recombinant protein more efficiently. The targeted deletion regions were ISs, K-islands, flagellar genes, ciliated genes, and lipopolysaccharide genes, which were identified according to the *E. coli* data bank.

#### 3.4.2. Growth

The growth rate of MS56 was equivalent to that of the wild-type strain in LB [65]. However, the growth of MS56 in the minimal medium presented conflicting results, that is, it was either 1.6-fold faster [65] or significantly slower [55] than that of the wild-type strain. It suggested that the deleted genes might play a role in the nutritional requirements and may be sensitive to slight differences in culture conditions including media compositions.

### 3.5. CDΔ3456

#### 3.5.1. Design Principles

CDΔ3456 was another MG1655-derived genome-reduced strain [66], which lacked 313.1 kb of the genomic sequence. This strain was also constructed for determining the minimum set of genes, as did for Δ16. The Tn5-targeted *cre/loxP* excision system was used for the construction. Tn5 transposons carrying *loxP* sequences were randomly placed in the *E. coli* chromosome, and the region between the two *loxP*s was deleted by Cre recombination. Six strains with a single deletion region were constructed, and CDΔ3456 was constructed by deleting four out of the six regions.

#### 3.5.2. Growth

There is little information concerning the growth of CDΔ3456. The only study on it reported that the strain showed a growth rate equivalent to that of the wild-type strain when cultured in LB [66].

## 4. Aims and Additional Characteristics of Genome-Reduced Strains

Differentiation in the phenotypic properties of the reduced genomes somehow resulted from the different design principles and purposes. The genome-reduced *E. coli* strains of MGF-01, MDS69 and MS56 were constructed for the purpose of achieving simple and highly controllable cells without nonessential genomic regions, and the strains of Δ16 and CDΔ3456 were constructed to determine the minimum components essential for maintaining cellular activities. Whether and/or how such differentiation affected the other characteristics is discussed as follows.

### 4.1. Genome Stability

In addition to the growth of the cell population, the stability of the genome was another important factor for the genome-reduced *E. coli* strains to be used for the aim of metabolite production. It was reported that the mutations caused unexpected disadvantages and reduced substance productivity [44,45]. Since the ISs were removed from MDS, the stability of the reduced genome of MDS was supposed to be improved. To verify this assumption, the genome-reduced strain MDS41 and the wild-type strain MG1655 were cultured in the minimum medium containing salicylic acid as the only carbon source [59]. To metabolize salicin, the *bgl* operon was required to be activated. The activation of *bgl* operon can be achieved by the insertion of ISs into the promoter region [67]. The results showed that the activation rate in MDS41 was only ~8% of that in MG1655, indicating that the IS-related mutation rate was significantly decreased due to genome reduction [59]. Similarly, MS56 showed higher stability for the production of the recombinant proteins than that of MG1655 [65], owing to the disappearance of ISs. The improved stability was considered as advantageous for industrial production, although the capacity of the foreign genes (plasmids) for production might be reduced [68].

The non IS-related mutation rate was equivalent in both strains [59]. On the other hand, the chemostat culture of MDS42 and MG1655 in the minimal medium M9 accumulated an equivalent number of mutations [69]. In addition, the mutation rate of MGF-01 was increased, which was triggered by genome reduction [56]. Thus, genome reduction did not always lead to improved stability. From the view point of industrial production, the improved stability of the genome was advantageous in offering a stable cellular environment; however, the deleted sequences, such as ISs, were thought to be essential for environmental adaptation, e.g., the new insertion of an IS element in MDS42 improved its fitness [70].

### 4.2. Genome Minimization

Genome minimization was discussed for decades, and it was estimated that the minimum gene set contained at least 256 genes, which was determined by the comparative genomic analysis of *Mycoplasma genitalium* and *Haemophilus influenzae* in 1996 [71]. However, the essential genes, which were commonly defined as the genes whose single deletion was lethal, were highly variable even within the same species; comparative genomic analysis was insufficient to address this question [72]. Intensive experimental challenges were performed, such as the systematic construction of the single-gene knockout *E. coli* strains, the Keio collection [73]. The single-gene knockout experiment identified ~ 300 essential genes, however, the deletion of multiple non-essential genes could be lethal [74]. It indicated that the minimal gene set still requires further investigation [75]. The so-called top-down and bottom-up approaches were recently proposed to explore the minimal gene set essential for sustainable life [76]. The top-down approach was to remove the nonessential genome regions of the living cells, that is, genome reduction, which was commonly used in the bacteria of *E. coli* and *B. subtilis* [35]. For instance, the largest deletion of genomic sequence in *E. coli* produced the strain Δ33a, in which 38.9% of the genomic region was deleted from the wild type strain MG1655 [77]. A representative study of genome minimization using the bottom-up approach was the construction of JCVI-syn3.0, which was a *Mycoplasma capricolum* cell carrying a synthetic genome smaller than any *mycoplasma* genomes [3]. The recently reported chemical synthesis of the entire genome of *E. coli* MDS42 using 61 codons was a remarkable achievement of the bottom-up approach for the artificial cells [38]. Accordingly, the expectation of being able to construct the minimal genome artificially from scratch was realistic. However, even if the genome could be fully synthesized, the design principles remained unknown. The top-down approach was thought to be effective, as the trajectory from the wild-type to the minimum genome was supposed to be traceable; in addition, the connection between genome reduction and cellular phenotype could be investigated. The genomic sequence essential for a sustainable life remains to be addressed by genome reduction. Recently, the in silico design of genome reduction according to the metabolic models became applicable [78], which allowed the genome reduction to be more practical and controllable.

## 5. Proposals for Further Challenges

### 5.1. Concerns for the Environmental Factors

Genome reduction without a growth decrease is a key point for both the minimal genome and applications in synthetic microbiology, because the growth rate affected the cell’s physiological state, consequently contributing to metabolite productivity [79]. In addition to the genomic sequence itself, the external environment, e.g., media conditions, requires attention. MGF-01 was reported to have an equivalent growth rate to W3110 when cultured in M9 [53], but another study reported that MGF-01 grew slower than W3110 when cultured in the minimal medium M63 [7]. The growth rate of MGF-01 declined with increasing genome reduction, and the growth rate decrease was more significant under malnutrition conditions [7]. MS56 presented a declining growth rate in the minimal medium M9 but maintained a growth rate equivalent to that of MG1655 in the rich medium LB [55]. The results strongly indicated that the growth decline caused by the genome reduction could be compensated for by the external conditions. In wild nature, the genomes of the symbiotic bacteria were largely reduced during evolution, which was probably owing to nutritional supplementation from hosts [47,80]. Thus, it is more reasonable to rely on external nutrition than to keep the genes that synthesize nutrition on their own when external nutrition can be obtained. In addition to growth rate, genome stability might be affected by medium composition. Although the clean genome MDS42 was constructed for stability, it showed mutation rates similar to that of the wild-type strain under the minimum medium [69]. It was assumed that the reduced genome might be more sensitive to medium composition. The malnutrition induced the stress-induced mutagenesis, which reduced genome stability.

### 5.2. Approaches for Experimental Evolution

It was assumed that modern *E. coli* has the optimal genome due to its 3.7 billion years of evolution since the origin of the microorganism [81]. Genome reduction could be stressful for *E. coli*, so that the growth rate would be decreased. Experimental evolution was an efficient approach to raise the growth fitness of bacterial cells, because it simulated evolution by reproducing the conditions in which the better-adapted mutated cells were selected as the generation that continued. Experimental evolution has been widely employed in the studies on the mechanisms of adaptive evolution. In addition to long-term evolution [82], the adaptive evolution to toxic environments [83,84,85], various temperatures [86,87,88] and pH conditions [89], has been reported. These studies commonly showed the gradually increased growth rates, which might be caused by beneficial mutations [90,91,92]. This approach was practical for the reduced genomes as well (Figure 2B). During the serial transfer, MGF-01 recovered its growth by approximately 400 generations [56], and MS56 matched the growth rate of MG1655 after the experimental evolution of ~800 generations [55]. Chemostat culture of MDS42 and MG1655 for 21 days led to a significant increase in MDS42 growth but not MG1655 growth [57]. These results clearly demonstrated that the experimental evolution was efficient in increasing the growth of reduced genomes for further reduction. The reasons for the increased growth rates caused by the experimental evolution are still unclear; the increase in translation efficiency [55] or the increase in fast-growing cells in the population [57] have been proposed as explanations. In addition, the consequence of experimental evolution has often been linked to the homeostasis of biological systems. The metabolic system, which was disrupted by gene knockout, was reported to be re-optimized by the experimental evolution [93]. Besides, the gene expression disturbed by the changes in nutrient sources or temperature has tended to be close to the original expression pattern [94]. These findings suggest that experimental evolution has helped living cells carrying either a complete or a reduced genome to reach a steady physiological state and/or proper growth fitness. The genome reduction associated with experimental evolution may allow us to achieve a more efficient cell factory as well as the minimal genome.

## Supplementary Materials

Supplementary materials can be found at https://www.mdpi.com/2076-2607/8/1/3/s1.
