# Supplementary material for: Experimental Challenges for Reduced Genomes: The Cell Model Escherichia coli
Source: Microorganisms. 2019 Dec 18;8(1):3. doi: 10.3390/microorganisms8010003 (PMC7022904; doi:10.3390/microorganisms8010003)
Supplement: Supplementary file 1 [file microorganisms-08-00003-s001.pdf]

Experimental Challenges for Reduced Genomes: The Cell Model *Escherichia coli*

Masaomi Kurokawa and Bei-Wen Ying

Table S1. Growth properties and culture conditions of genome-reduced *E. coli* strains. Strains and Genome del. indicate the names of the genome-reduced *E. coli* strains used in the original papers and the deleted length of the genomic sequences, respectively. The percentages of the reduced genome sizes are noted in the brackets. Growth rate and Growth max. represent the changes in the growth rates and in the maximal OD caused by the genome reduction, respectively. The ratios of growth changes are indicated, if applicable. The conditions for cell culture are described in Media and Culture vol., which indicate the medium compositions and the volumes, respectively. Refs represents the reference numbers of the original studies cited in the main text. n.d., no data.

	Genome del.	Growth rate	Growth max.	Media	Culture vol.	Refs.
MGF-01	1.03 Mb (22%)	Equivalent	Higher, 150%	M9 with 10 mg/L $FeSO_4$, 20 g/L $CaCO_3$ and 10 g/L glucose	20 mL, flask	[53]
		Decreased, 53%	Lower, 82%	M63	200 μL, 96-well microplate	[7]
		Decreased, 83%	Lower, 96%	M63 with 0.02 mM Tyr and 0.05 mM 19 amino acids	200 μL, 96-well microplate	[7]
		Decreased, 92%	Lower, 92%	LB	200 μL, 96-well microplate	[7]
		Increased	Higher	CSL	800 mL in 2-L jar fermenter	[51]
DGF-298	1.67 Mb (35.8%)	Increased	Higher	CSL	800 mL in 2-L jar fermenter	[51]
MDS12	376.2 kb (8.1%)	Equivalent	Higher, 110%	LB	100 mL	[58]
		Equivalent	Higher, 110%	M9 with glucose and 0.5% casamino acids	100 mL	[58]
		Equivalent	Higher, 110%	M9 with glucose	100 mL	[58]
MDS41	663.6 kb (14.3%)	Equivalent	n.d.	minimal medium	1.5 L in 2-L jar fermenter	[59]
MDS42	663.3 kb (14.3%)	Equivalent	n.d.	MOPS minimal medium	50 mL in 250-mL flask	[59]
		Equivalent	n.d.	LB	50 mL in 250-mL flask	[59]
		Equivalent	n.d.	Defined Rich medium	50 mL in 250-mL flask	[59]
		Decreased, 75%	n.d.	M9 with 1 mM glucose and 0.2 % casamino acids	microplate	[57]
MDS69	939.5 kb (20.3%)	Decreased, 83%	n.d.	LB + 15% DMSO	50 μL in 384-well microplate	[60]
Δ16	1.38 Mb (29.7%)	Decreased, 58%	n.d.	Antibiotic Medium 3	n.d.	[8]
MS56	1068 kb (23%)	Increased, 160%	n.d.	minimal medium (no details)	n.d.	[65]
		Equivalent	n.d.	LB	n.d.	[65]
		Decreased, 37%	n.d.	M9 with glucose	n.d.	[55]
		Decreased, 87%	n.d.	LB	n.d.	[55]
CDΔ3456	313.1 kb (6.8%)	Equivalent	n.d.	LB	n.d.	[66]

Table S2. Media used for growth assays of the reduced genomes. The compositions of six media (in bold) used to test the growth of the genome-reduced *E. coli* strains are summarized. References indicates the reference numbers of the original papers cited in the main text. *, 1M $MgSO_4$; **, $MgSO_4 \cdot 7H_2O$.

	**M9 buffer**		**M9**		**M63**		**Antibiotic Medium 3**		**CSL**		**MOPS MM**	
Na_2HPO_4	6	g/L	47.5	mM								
K_2HPO_4					62	mM	3.68	g/L	23.4	g/L	1.32	mM
KH_2PO_4	3	g/L	22.04	mM	39	mM	1.32	g/L				
NaCl	5	g/L	8.56	mM			3.5	g/L	1.6	g/L	50	mM
NH_4Cl			18.7	mM							9.52	mM
$CaCl_2$			0.1	mM							0.5	μM
$CoCl_2$											0.3	μM
$MnCl_2$											0.8	μM
$MgCl_2$											0.523	mM
$MgSO_4$	1	mL/L*	2	mM	0.2	mM			1.67	g/L**		
$FeSO_4$	10	mg/L			1.8	μM					0.01	mM
$(NH_4)_2SO_4$					15	mM			9.4	g/L		
K_2SO_4											0.276	mM
$CuSO_4$											0.1	μM
$ZnSO_4$											0.4	μM
$(NH_4)_6(MO_7)_{24}$											0.03	μM
thiamine-HCl					15	μM			7.8	mg/L		
$CaCO_3$	20	g/L										
H_3BO_3											4	μM
glucose	10	g/L	2	g/L	22	mM	1	g/L	30	g/L		
beef extract							1.5	g/L				
yeast extract							1.5	g/L				
peptone							5	g/L				
corn steep liquor									31.3	mL/L		
soy peptone									7.8	g/L		
nicotinic acid									7.8	mg/L		
threonine									31.2	mg/L		
tryptophan									31.2	mg/L		
leucine									31.2	mg/L		
MOPS											40	mM
Tricine											4	mM
References	[52, 53, 58]		[55]		[7, 54]		[8]		[51]		[59, 61]	
